# Intractable or persistent hiccups treated with extracranial acupuncture: Two case reports

**DOI:** 10.1097/MD.0000000000020131

**Published:** 2020-05-15

**Authors:** Zong-wang Zhang, Chang-xiang Gong

**Affiliations:** a Department of Anesthesiology, Shandong Liaocheng People's Hospital, Liaocheng; b Beijing Guoao Psychological Hospital, Beijing, China.

**Keywords:** acupuncture, case report, intractable hiccup, traditional Chinese medicine, treatment

## Abstract

**Rationale:**

Hiccups are a common clinical symptom, and persistent hiccups and intractable hiccups severely impair the individual's quality of life. To date, there has been no effective treatment specifically for hiccups. Herein, we report 2 cases with intractable or persistent hiccups that were successfully treated with extracranial acupuncture.

**Patient concerns:**

The first case is a 46-year-old woman who presented with a 7-year history of intractable hiccups that had worsened over the past 3 years. She also complained of chest tightness, dyspnea, palpitations, dreaminess, dysphoria, intolerance of cold, and hypohidrosis. The second case is a 75-year-old man who presented with a 7-day history of persistent hiccups and hematemesis for 3 hours. The patient's persistent hiccups were treated using traditional Chinese acupuncture, but the patient reported no remarkable benefit.

**Diagnoses:**

They were diagnosed as intractable or persistent hiccups.

**Interventions:**

They were treated with extracranial acupuncture.

**Outcomes:**

The hiccups completely disappeared. During the follow-up period, the hiccups did not reappear.

**Lessons:**

According to neural balance theory, an episode of the hiccups is caused by an imbalance of the nervous system. Extracranial acupuncture in the area adjacent to the external occipital protuberance affects the intracranial nervous system, which can effectively control the hiccups. Our study provides a new approach to the treatment of hiccups.

## 1 Introduction

Hiccups are a common clinical symptom, characterized by an involuntary spasm of the diaphragm that causes a sudden inspiration with closure of the glottis. An episode of the hiccups lasting in duration anywhere from 48 hours to 1 month is defined as persistent hiccups; an episode that lasts longer than 1 month is defined as intractable hiccups.^[1]^ Intractable hiccups are attributed to structural or functional disorders of the medulla or the respiratory muscles, metabolic and/or endocrine deficiencies, drugs, anesthesia, and/or emotional abnormalities.^[2]^ The hiccups is generally a self-limited disease that can resolve spontaneously without any treatment. However, intractable hiccups may cause dehydration, insomnia, depression, and digestive disease, and sometimes severely impair the individual's quality of life.^[3]^ To date, no effective treatment has been identified.

**Figure 1:**
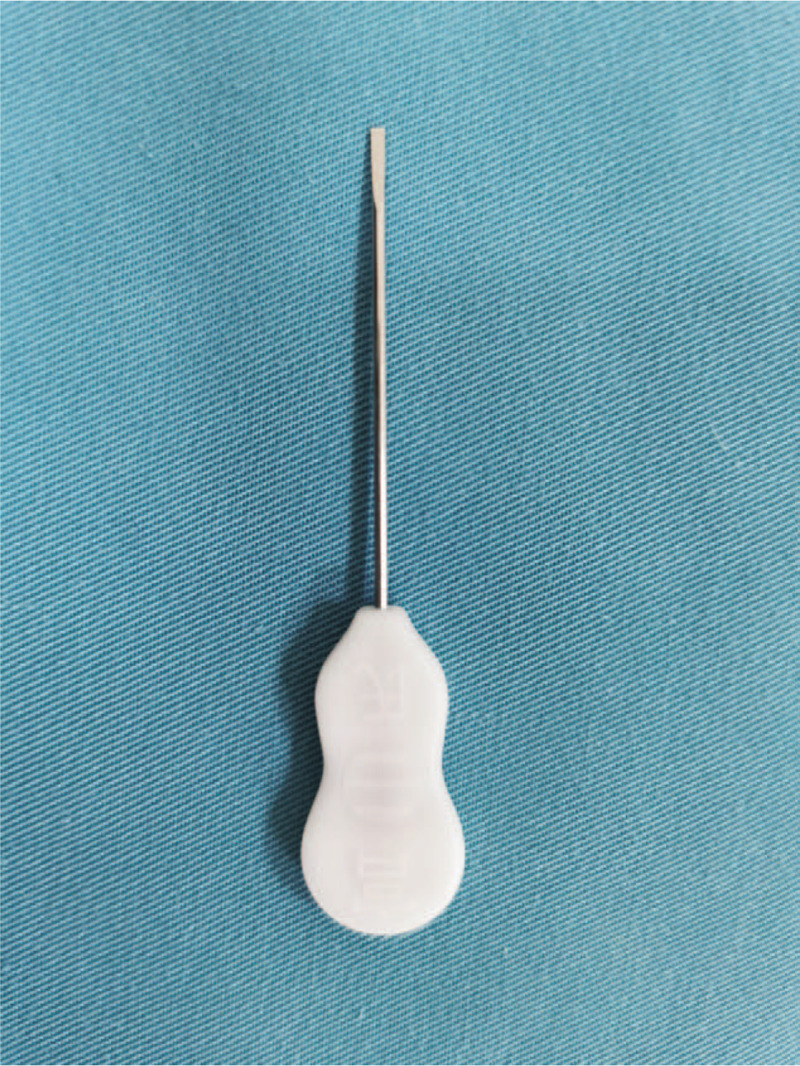
“Brain needle” used for extracranial acupuncture.

Dr Gong has been engaged in clinical research on pain for >20 years. Over the course of many years of clinical experience, he found that stimulating the external occipital protuberance and its nearby bone tissue using conventional acupuncture techniques may effectively treat nervous system dysfunction. Extracranial acupuncture therapy, originally proposed by Dr Gong Changxiang, is also known as Gong nerve regulation technology. Professor Zhou Liqun at Beijing University of Traditional Chinese Medicine concluded that Gong theory, which adopts traditional acupuncture methods from traditional Chinese medicine, also reflects the principles of neuroscience. The site of stimulation is the head, so this treatment paradigm is known as the “brain needle” (Fig. 1). Over the past 3 years, extracranial acupuncture has successfully cured or relieved functional symptoms such as pain, insomnia, depression, and neurodermatitis. However, this therapy has never been reported in the literature. Herein, we report 2 cases with intractable or persistent hiccups that were successfully treated with extracranial acupuncture. This study was approved by the local Ethics Committee. Written informed consent was obtained from the patients.

## 2 Case presentation

### 2.1 Case 1

A 46-year-old woman presented with a 7-year history of intractable hiccups that had worsened over the past 3 years. Seven years ago, the patient had experienced an episode of depression accompanied by occasional hiccups after social disputes. In such cases, the hiccups resolved spontaneously without treatment. Fifteen days after the depressive episode, the hiccups began to occur frequently, especially at night; each episode lasted 3 to 4 hours, preventing the patient from sleeping. The patient's hiccups were induced by angry emotions and cold or rainy weather. After 30 days, these hiccup attacks became more frequent; still, each episode lasted 3 to 4 hours. Each attack was accompanied by chest and abdominal convulsions and pain as well as asphyxia. The patient felt frightened. Her hiccups were sometimes relieved by pinching the skin between her eyebrows. The patient subsequently noted that her episodes of hiccups could also be temporarily relieved by pinching the lumbosacral skin.

The patient was admitted to the local hospital. Gastroenteroscopy showed no abnormalities; spasmolysis and sedation drugs were prescribed, but they provided no benefit. Then, the patient was treated with traditional Chinese medicine (>1000 doses), but this exacerbated her hiccups. Physical examination revealed a pale complexion, dull expression, and continuous attacks of hiccups. During each attack, the patient was forced to bend down. She complained of chest tightness, dyspnea, palpitations, dreaminess, dysphoria, intolerance of cold, and hypohidrosis. She preferred hot drinks. The patient's blood pressure, heart rate, respiration, pulse, and other vital signs were all normal. After the patient had provided written informed consent, she underwent extracranial acupuncture treatment for the first time.

**Figure 2:**
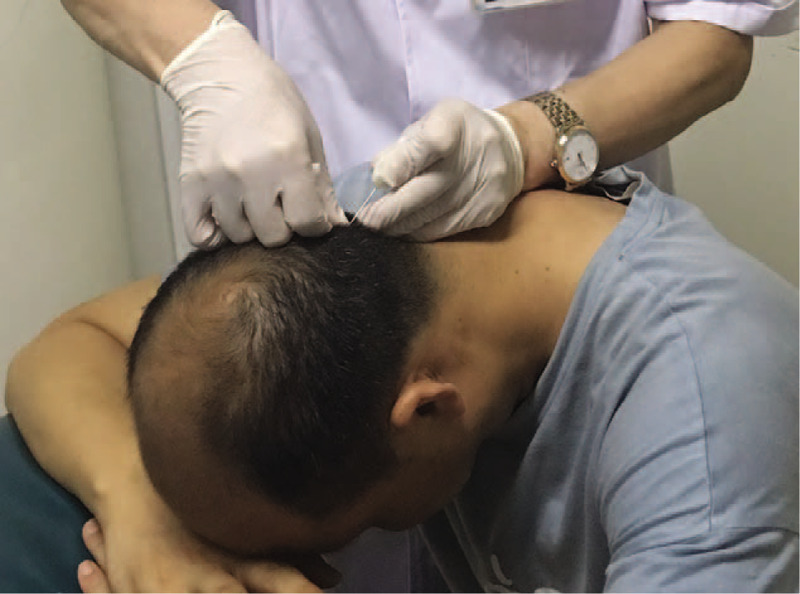
Protocol used for extracranial acupuncture.

A sitting position was adopted, with the patient's hands folded on the treatment bed. The patient's head was bent forward, with hers forehead against the back of her hands. The acupuncture point was located 0.5 cm distal to the point of attachment between the occipital ligament and the external occipital protuberance. After routine skin disinfection, the distance between the skin and the bone surface was evaluated with one hand holding the needle and the other hand pressing the skin at the acupuncture point. Then, the tip of the needle was placed against the skin and quickly inserted. After the percutaneous puncture, the needle angle was adjusted to ensure that the needle was perpendicular to, and in contact with, the bone surface. Then the acupuncturist's auxiliary hand quickly squeezed the lower part of the needle, until it had penetrated approximately 1 mm into the bone. Once the needle had been clamped by the bone, the needle was pulled out (Fig. 2). Sterile gauze was applied to the acupuncture point with pressure for about 5 minutes. Ten minutes after the treatment, the patient's symptoms (including chest tightness, dyspnea, and hiccups) had completely disappeared. The patient was relaxed and in a comfortable mood. The first acupuncture treatment gave the patient more confidence. The patient's subsequent treatment included 10 acupuncture sessions (once daily), with the acupuncture point repositioned 1.5 to 2 cm distally toward the top of the head each time. After the first therapeutic course, the duration of the patient's nighttime hiccups decreased to 1 hour, and the quality of her sleep improved. The duration of the patient's daytime episodes of hiccups decreased to 2 hours. The patient then underwent a second course of treatment after a 7-day interval. During this period, acupuncture was performed every 2 days. After the second course of treatment, the patient experienced an episode of hiccups every other day but reported no continuous attacks. The hiccups had resolved completely by the time the patient had completed 3 courses of treatment. During a follow-up period of 1 year, the hiccups did not return.

### 2.2 Case 2

A 75-year-old man was admitted to the Department of Gastroenterology with a 7-day history of persistent hiccups and hematemesis for 3 hours. A diagnosis of Mallory-Weiss syndrome was made. Seven days prior to admission, the patient caught a cold and experienced an episode of hiccups (20 per minute) that lasted for 1 hour. The patient's hiccups were transiently relieved by drinking water but aggravated by eating. No specific treatment was given. Three hours before admission, the patient's hiccups began to be accompanied by nausea and hematemesis (about 15 mL per episode). There was no melena, dizziness, fatigue, ataxia, syncope, palpitation, chest or abdominal pain, or abdominal distension. The patient had a 15-year history of hypertension and a 7-year history of diabetes, and he regularly took oral nifedipine and subcutaneous insulin. Fourteen years earlier, the patient had undergone coronary artery bypass surgery; since then, the patient had taken oral isosorbide mononitrate. Three years prior to presentation at our hospital, the patient had undergone surgical resection of nucleus pulposus caused by lumbar disc herniation; he recovered rapidly from the procedure. One year prior to presentation at our clinic, the patient had been implanted with a stent because of cerebrovascular stenosis; since that point, he had been prescribed oral clopidogrel. On admission, the patient's body temperature was 36.9 °C; heart rate was 96 bpm; respiratory rate was 22 breaths per min; blood pressure was 110/80 mmHg. The results of the patient's laboratory tests were as follows: white blood cells 6.26 × 10^9^/L, hemoglobin 115 g/L, fibrin 3.22 mg/L, prothrombin 12.3 second, and international standardized ratio 1.15.

After admission, a diagnosis of acute erosive-hemorrhagic gastritis was suspected. Oral omeprazole, hemocoagulase, octreotide acetate, and vitamins were administered. The patient's persistent hiccups were treated using traditional Chinese acupuncture, but there were no remarkable benefits. Then, extracranial acupuncture was performed according to the methods described in case 1. The acupuncture point was located 0.5 cm distal to the point of attachment between the occipital ligament and the external occipital protuberance. Immediately after the first extracranial acupuncture, the patient's hiccups disappeared; they did not recur thereafter. During a 6-month follow-up period, the patient remained asymptomatic.

## 3 Discussion and conclusions

Hiccups are a common symptom in patients with gastrointestinal or central nervous system diseases. More than 20% of patients with Parkinson disease and 10% of patients with gastroesophageal reflux have recurrent hiccups.^[2,3]^ The pathogenesis of hiccups may be associated with a neural reflex that involves the vagus nerve, phrenic nerve, and/or sympathetic fibers at the levels of T6 to T12. The neural centers thought to be involved include the C3 to C5 spinal cord, the respiratory center of the medulla oblongata, the reticular structure of the brainstem, and the hypothalamus. The efferent nerve thought to be involved is the phrenic nerve.^[4]^

Dopamine and gamma-aminobutyric acid (GABA) neurotransmitters play a role in regulating the function of the central nervous system.^[4]^ Glottis closure is controlled by the recurrent laryngeal nerve. Therefore, any factors that stimulate the afferent nerves listed above, the central nervous system, or efferent nerves can lead to hiccups. Common causes of hiccups include: gastric distension caused by satiety or carbonated drinks; irritation of the digestive tract or respiratory tract by chili, alcohol or tobacco; excessive excitement; anxiety; gastrointestinal disease; cerebrovascular disease; brain tumor; brain trauma; cardiovascular disease; electrolyte imbalance; uremia; hyperglycemia; and drug-related adverse effects.^[1]^

The intractable hiccups in case 1 may have been caused by tension, anxiety, and/or depression associated with social disputes. The persistent hiccups in case 2 may be attributed to infection of the upper respiratory tract and gastric dilation, or to cardiovascular and/or cerebrovascular disease.

The conventional treatment for persistent or intractable hiccups includes drugs that act on dopamine and GABA receptors (such as chlorpromazine, metoclopramine, and baclofen), anticonvulsants (such as gabapentin), and calcium channel blockers (such as nifedipine).^[5]^ It has been reported that acupuncture may be effective in controlling hiccups.^[6]^ Positive pressure ventilation, hypnosis, surgical intervention, and the stimulation of hiccup-related nerve reflex arcs have also been highlighted as potential treatments for hiccups.^[7]^ However, all of the above methods have limitations; to date, no specific therapy has been found to be effective.

Considering the fact that persistent or intractable hiccups are caused by neural reflex dysfunction, Dr Gong innovatively put forward the neural balance theory, arguing that hiccups represents a local symptom of functional imbalance in the nervous system. In case 1, the hiccups may have been caused by abnormal central nervous system function as a result of anxiety and depression after disputes; in case 2, the hiccups may have been caused by abnormal peripheral nerve function. Additionally, because the hiccups persisted for a long time, the symptom of hiccups may be learned by the brain; involvement of neural circuits in the brain transforms the condition into central hiccups. The central nervous system plays an important role in the treatment of hiccups, even when caused by abnormal peripheral nerve function, because the central nervous system coordinates excitation and inhibition of the peripheral nervous system. Gong extracranial acupuncture may therefore affect the intracranial central nervous system and then exert inhibitory effects on the peripheral nerves. This is a plausible explanation for the effectiveness of extracranial acupuncture in the treatment of hiccups. As nerve tissues, bone tissues have short-term and long-term memory functions. There is an interactive neural network among bone cells and neurons.^[8]^ Intracranial nerve tissue may be affected by the stimulation of extracranial bone tissue. However, the specific mechanism remains to be elucidated.

According to the neural balance theory, the hiccups is caused by an imbalance of the nervous system. Gong extracranial acupuncture acts through bone tissue in the external occipital protuberance on the intracranial nervous system, which can effectively control the hiccups. This case report provides a new approach to the treatment of hiccups.

## Author contributions

Chang-xiang Gong put forward the theory of neural balance, invented the method of brain acupuncture, and was responsible for the treatment of case 1. Zong-wang Zhang is responsible for the treatment of case 2 and the writing and publication of papers. All of the authors have read and approved the manuscript.
